# From Trace to Pure: Pilot-Scale Scandium Recovery from TiO_2_ Acid Waste

**DOI:** 10.1021/acssuschemeng.2c06979

**Published:** 2023-04-06

**Authors:** Sebastian Hedwig, Bengi Yagmurlu, Edward Michael Peters, Victor Misev, Dirk Hengevoss, Carsten Dittrich, Kerstin Forsberg, Edwin C. Constable, Markus Lenz

**Affiliations:** † FHNW, Institute for Ecopreneurship, Hofackerstrasse 30, 4132 Muttenz, Switzerland; ‡ Department of Chemistry, University of Basel, Mattenstrasse 24a, 4058 Basel, Switzerland; § TU Clausthal, Institute of Mineral and Waste Processing, Recycling and Circular Economy Systems, Walter-Nernst-Str. 9, 38678 Clausthal-Zellerfeld, Germany; ∥ MEAB Chemie Technik GmbH, 52068 Aachen, Germany; ⊥ Department of Chemical Engineering, KTH Royal Institute of Technology, 100-44 Stockholm, Sweden; # Department of Environmental Technology, Wageningen University, Bornse Weilanden 9, 6700 AA Wageningen, The Netherlands

**Keywords:** critical raw material, secondary source TiO_2_ pigment production, chloride route, nanofiltration, solvent extraction, antisolvent crystallization, scandium, cost

## Abstract

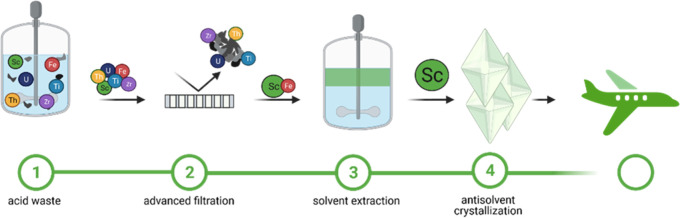

Scandium (Sc), declared a critical raw material in the European Union (EU), could face further supply issues as the EU depends almost entirely on imports from China, Russia, and Ukraine. In this study, a tandem nanofiltration-solvent extraction procedure for Sc recovery from titania (TiO_2_) acid waste was piloted and then augmented by antisolvent crystallization. The new process, comprising advanced filtration (hydroxide precipitation, micro-, ultra-, and nanofiltration), solvent extraction, and antisolvent crystallization, was assessed in relation to material and energy inputs and benchmarked on ScF_3_ production. From ∼1 m^3^ of European acid waste containing traces of Sc (81 mg L^–1^), ∼13 g of Sc (43% yield, nine stages) was recovered as (NH_4_)_3_ScF_6_ with a purity of approximately 95%, demonstrating the technical feasibility of the approach. The production costs per kilogram of ScF_3_ were lower than reported market prices, which underscores a competitive process at scale. Although a few technical bottlenecks (e.g., S/L separation and electricity consumption) need to be overcome, combining advanced filtration with solvent extraction and antisolvent crystallization promises a future supply of this critical raw material from European secondary sources.

## Abstract 2

Piloted scandium separation from TiO_2_ production waste using a combination of advanced filtration techniques, solvent extraction, and antisolvent crystallization.

## Introduction

Supply chains are the backbone of the economy. However, their resilience is increasingly challenged by social, environmental, and geopolitical factors, potentially leading to disruption and, consequently, economic damage. Herein, critical raw materials (CRMs) are a key factor, as they are economically important but at risk in supply. To identify and counteract raw material criticality, the European Union (EU) has launched a raw material initiative, publishing a list of CRMs every three years since 2011.^1^ The rare earth metal scandium (Sc) has been included on the list since 2017 because of its applications in high-strength aluminum alloys and high-efficiency fuel cells.^1,2^ Used in aircraft engineering, up to 20% lighter airplanes could be built compared with today’s standards.^3^ Sc_2_O_3_ is vital for commercialized solid oxide fuel cells, which facilitates the direct conversion of hydrogen to electrical power.^3,4^

However, market acceptance has been low due to a severe lack of Sc supply and extremely high prices.^5^ Until recently, the majority of the supply came from China (66%), with Russia (26%) and Ukraine (7%) as other suppliers.^6^

The underdevelopment of Sc production can be partially attributed to the scarcity of Sc ores. Sc has a low affinity to common ore-forming anions and, thus, is widely dispersed in the lithosphere.^7^ Consequently, Sc recovery from secondary sources, where it is concentrated, is a compelling notion. One example of secondary sources is waste from the chloride route for white pigment (TiO_2_) production. This two-stage process is responsible for 3–4 Mt a^–1^ or approx. 50% of the global TiO_2_ supply.^8^ Rutile or titania-rich starting materials are converted into volatile TiCl_4_ using Cl_2_ and coke. After separation, TiCl_4_ reacts with O_2_ and pure TiO_2_ is obtained, whereas Cl_2_ is recycled. Impurities accompanying the starting materials are washed out in the scrubber water.^9^ These impurities contain HCl (approx. 15%), unreacted ore, coke, and a variety of metal chlorides. Sc has been reported to be present in the range of several hundred ppm.^9,10^

Some approaches have been developed to recover Sc. Conventionally, solvent extraction (SX) is used, followed by precipitation as a hydroxide or oxalate salt.^11,12^ After calcination at temperatures in the range of 700–800 °C, Sc_2_O_3_ is obtained.^13^ The oxide is then converted to ScF_3_ using hydrofluoric acid.^14^ Afterward, metallothermic reduction of ScF_3_ is conducted to produce Sc metal or Al–Sc alloys.^14−16^

Remmen et al. presented nanofiltration (NF) using tailor-made layer-by-layer assembled membranes (LbL membranes) as a viable option, retaining most of the Sc while partially depleting impurities.^3^

We showed in our previous study that the combination of NF and SX can be successfully utilized to produce a strip liquor containing >97% pure (NH_4_)_3_ScF_6_ from genuine TiO_2_ acid waste.^10^ Peters et al. reported the further purification of such Sc strip liquors using antisolvent crystallization (ASC) with ethanol to yield >98.7% pure (NH_4_)_3_ScF_6_.^17^ It was also reported that the metals are usually present in the solid product in relative proportions that reflect their abundance in the strip liquor.^18^ Furthermore, studies on the solubility of (NH_4_)_3_ScF_6_ in NH_4_F solutions and NH_4_F-alcohol mixtures were published.^19^ The ammonium metal fluorides of Fe and Al were shown to exhibit considerably lower solubilities than (NH_4_)_3_ScF_6_, while (NH_4_)_3_ZrF_7_ exhibited comparable solubility to (NH_4_)_3_ScF_6_, in NH_4_F-alcohol mixtures.^18^ Further studies showed the importance of supersaturation control on the quality of the product crystals and that trade-off exists between product quality and productivity.^20^

However, a discrepancy was found between the dimensions of the prospected Sc recovery route and the volume of waste generated. Therefore, this study aimed to upscale the previously presented seven-stage NF-SX process by treating ∼1 m^3^ of real TiO_2_ acid waste. In addition, the final solid product was synthesized, and the quality was enhanced by ASC. The newly developed procedure was assessed in terms of the material and energy costs required to produce 1 kg of ScF_3_ as the closest marketable product in the Sc supply chain.

## Materials and Methods

### Chemicals and Materials

Acid waste was obtained from a TiO_2_ producer in The Netherlands. NaOH solution (30% w/w) for pH adjustment was provided by GETEC PARK.SWISS, Switzerland.

HCl (37% w/w, laboratory grade, PANREAC QUIMICA S.L.U., Spain), NH_4_F (reagent grade, Merck, Germany), D2EHPA (Lanxess, Germany), N1923 (HalloChem, China), and dearomatized kerosene (Exxsol D80, ExxonMobile, Germany) were used for SX.

Analytical-grade ethanol (99.95% v/v) for the ASC experiments was purchased from VWR, Sweden.

### Analytical Methods

#### Triple Quadrupole Inductively Coupled Plasma Mass Spectrometry (QqQ-ICP-MS)

Samples were diluted using nitric acid (3% w/w) and an autodilution system (Simprep, Teledyne Cetac Technologies). Thereafter, they were analyzed using QqQ-ICP-MS. The analysis was performed on an 8800 QqQ-ICP-MS system (Agilent, Switzerland) using general-purpose operational settings. Quantification was performed via multielement standards (0–50 ppb, seven points). To account for matrix effects, ^103^Rh was used as the internal standard. To quantify ^23^Na^+^, ^52^Cr^+^, ^55^Mn^+^, ^56^Fe^+^, ^60^Ni^+^, ^66^Zn^+^, ^89^Y^+^, ^137^Ba^+^, ^139^La^+^, ^140^Ce^+^, ^141^Pr^+^, ^146^Nd^+^, ^147^Sm^+^, ^153^Eu^+^, ^157^Gd^+^, ^159^Tb^+^, ^163^Dy^+^, ^165^Ho^+^, ^166^Er^+^, ^169^Tm^+^, ^172^Yb^+^, ^208^Pb^+^, ^232^Th, and ^238^U^+^, the ICP-MS was operated in single-quad mode using helium as the collision gas. Meanwhile, ^24^Mg^+^, ^27^Al^+^, ^39^K^+^, ^45^Sc^+^, ^47^Ti^+^, ^51^V^+^, and ^90^Zr^+^ were measured in triple-quad mass-shift mode using O_2_ as a reaction gas. ^7^Li^+^ concentration was determined using no-gas single-quad mode.

#### Inductively Coupled Plasma Optical Emission Spectrometry (ICP-OES)

Element concentrations in the ASC tests were analyzed by ICP-OES (iCAP 7400, Thermo Fisher Scientific Inc., USA). Supernatant samples were withdrawn and filtered (0.2 μm, polypropylene syringe filters) prior to dilution. HNO_3_ (3.45% v/v) was used for dilution.

#### Powder X-ray Diffraction (XRD) and Scanning Electron Microscopy (SEM)

Powder XRD spectra were recorded on a Siemens D5000 (Siemens AG, Germany) to examine the crystalline phases of the product. Micrographs were captured via SEM using a Philips/FEI-XL 30 series environmental scanning electron microscope (Philips, The Netherlands) to assess crystal size and morphology.

### Neutralization

For pH adjustment, an intermediate bulk container (IBC, 1 m^3^ volume) was equipped with an agitator (SR6, Simix, Germany) and NaOH dosing pumps (Vantage 5000, Verder, Germany). An exhaust air connection (Figure S1) was attached. The pH and temperature were measured using an inline sensor (Aquastick, Thermo Fisher Scientific Inc., The Netherlands). Caustic soda (30% w/w, 150 L) was successively added to the acid waste (800 L) under stirring until pH 1.5 was reached. The reaction mixture (950 L) was stirred for 24 h before settling for 48 h.

### Microfiltration (MF)

MF was carried out using a bag filtration unit (2-EF6-F, Eurowater, Germany; Figure S1) with two filtration bags (size 2, polypropylene, 1 μm nominal removal rate, 17 L volume). The filtration unit was fed by emptying the precipitation tank from top to bottom using a dip tube and a peristaltic pump (Vantage 5000, Verder, Germany) with a variable flow rate until the pressure reached 2 bar. Afterward, pressurized air (4 bar) was applied to further dewater the filter cake. The filter bags were emptied periodically (after 8, 15, 20, and 23 h) and reused until the filtration of the batch was completed. In total, 700 L of filtrate was separated from 250 L of hydroxide sludge.

### Ultrafiltration (UF) and NF

Both UF and NF were carried out in cross-flow operation mode using a modified filtration system (Osmo Inspector, Convergence, The Netherlands; Figure S1). For UF, 1812 spiral wound elements (UP150, Microdyn-Nadir, Germany, membrane area: 0.23 m^2^, MWCO: 150 kDa) were used. For batch UF (500 L), a transmembrane pressure (TMP) of 5–20 bar was applied at a cross-flow rate of 8 L min^–1^ and a *T* of 25 °C. The UF was stopped after 80% permeate recovery (400 L).

A 2540 spiral wound element (NanoPro A-3014, AMS Technologies, Israel; membrane area: 1.6 m^2^, MWCO: 400 Da) was used for NF. Prior to use, the module was compacted overnight by filtrating water (TMP: 15 bar, cross-flow rate: 8 L min^–1^, *T*: 25 °C). NF was operated in batch mode, aiming for a permeate recovery of 60%. The TMP was kept constant at 35 bar at a cross-flow rate of 8 L min^–1^. In total, 250 L was filtrated in five batches (50 L each) using the same membrane module without intermediate washing (Figure 3). Approximately 100 L of dark green concentrate was obtained after NF. Equations for calculating the concentration factor (*X)*, element (M) retention (*R*_M_), permeate flux (*J*_permeate_), and specific energy consumption (SEC) are given in the SI.

### Solvent Extraction

SX was conducted with NF concentrate (100 L) in a continuous countercurrent operation using 12 PVDF MEAB MSU-0.5 mixer-settler units (MEAB Chemie Technik GmbH, Germany) connected in series (Figure S2). The active mixer volume of the MSU-0.5 was 0.12 L, while the settler volume was 0.48 L with a loading surface area of 0.006 m^2^. The number of stages in each process step (extraction, scrubbing, and stripping) was determined by constructing the McCabe–Thile diagrams. Therefore, the respective solutions in each step were contacted with the organic solution with different phase ratios to obtain the equilibrium loading, scrubbing, and stripping curves (Figure 4).

To minimize Fe coextraction, Fe^0^ (1.5 g per liter) was added to the NF concentrate in a separate tank, reducing any Fe^3+^ to Fe^2+^. Afterward, Sc was extracted using 0.2 mol L^–1^ of D2EHPA with 0.05 mol L^–1^ of N1923 in D80 kerosene with a phase ratio of 4 (aqueous:organic). Coextracted impurities in the loaded organic were scrubbed with HCl (4 mol L^–1^) with a phase ratio of 0.1. The scrub liquor was recycled into the SX feed solution to eliminate Sc losses and control the pH for better Sc selectivity during SX. To remove entrained acid in the organic phase, which could lead to HF formation during stripping with NH_4_F, the scrubbed organic was washed with NaCl solution (2% w/w) with a phase ratio of 0.1. For Sc stripping, NH_4_F solution (3 mol L^–1^) was added to the washed organic with a phase ratio of 0.33, yielding an (NH_4_)_3_ScF_6_ solution. Finally, the organic was made to come in contact with HCl (2 mol L^–1^) with a 0.1 phase ratio to recondition the stripped organic phase and neutralize deprotonated D2EHPA.

### Antisolvent Crystallization

A strip liquor (pH = 5.74) after SX and stripping with NH_4_F solution (3 mol L^–1^) was used for the ASC tests. All tests were conducted in triplicate. To examine the Sc precipitation efficiency, ethanol (99.95%) was added all at once to aliquots of the strip liquor to reach final concentrations of 2, 4, 6, and 8 mol L^–1^, which corresponded to ethanol:strip liquor volumetric ratios of approximately 0.13, 0.31, 0.54, and 0.88, respectively. In addition, the precipitation efficiency of the other elements was examined at an ethanol concentration of 8 mol L^–1^. After ethanol addition, all suspensions were agitated at 500 rpm using a magnetic stirrer under ambient conditions for 1 h. The solid material obtained after crystallization at 8 mol L^–1^ of ethanol concentration was dried overnight under ambient conditions and used for further analysis.

## Results and Discussion

### Process Flow Scheme

**Figure 1:**
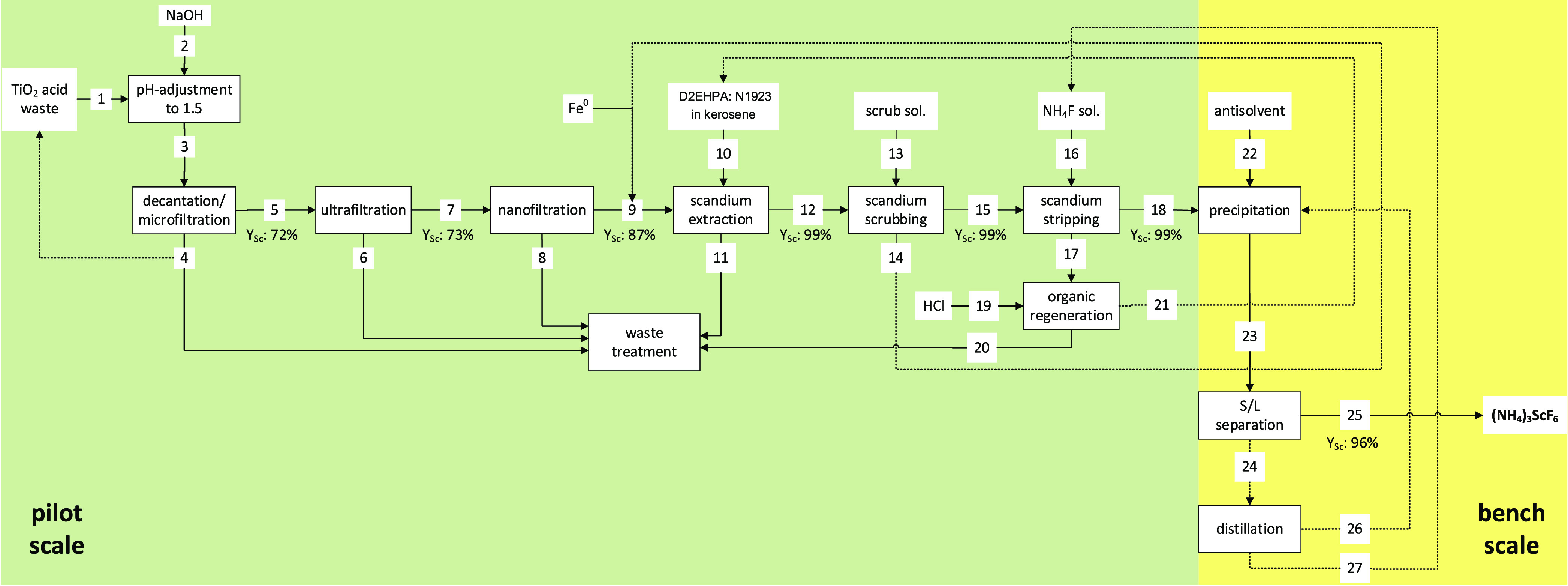
Block flow diagram of the scandium recovery process.

The process (Figure 1) was based on previous studies and comprised nine stages, excluding organic regeneration and final waste treatment.^10,17^ The first four stages (pH adjustment to NF) are summarized under the term “advanced filtration” (AF). Stages five to seven (Sc extraction, scrubbing, and stripping) are named SX. The last two stages (precipitation and S/L separation) are summarized under ASC. While AF and SX were tested on a pilot scale, ASC was conducted on a bench scale to optimize the parameters for recovering Sc from the strip liquor (Figure 1).

From the acid waste (stream 1), 12.6 g of Sc (43%) was recovered in the form of (NH_4_)_3_ScF_6_ (stream 25; Table 1). The total recovery yield after nine stages was higher than previously reported for bench-scale tests (36%, six stages)^10^ but still comparably lower than reported in other studies, such as Zhou et al. (68.6%),^21^ Chen et al. (90.34%),^22^ and Zhou et al. (95%).^23,24^ Major losses occurred in the early stages of AF (streams 1–9) within this study. Approximately half of the Sc (∼14.6 g) was lost after pH adjustment, MF and UF (streams 1–7). In contrast, virtually no losses occurred during SX (streams 9–18) and just minute amounts of Sc were lost (0.4 g, 3%) during ASC (streams 18 to 25). Therefore, considering the stages from NF to ASC, ∼84% yield was achieved (six stages, streams 7–25). Moreover, with respect to the latest five stages only (SX to ASC, streams 9–25), ∼97% of Sc yield was reached. Thus, while the yield of MF and UF leaves room for improvement, the yield of the other process stages was on par with the aforementioned studies.

**Table 1:** Mass Balance of the Sc Recovery Process Based on 100 L of NF Concentrate^a^

process step		pH adjustment			microfiltration		ultrafiltration		nanofiltration		Sc extraction			Sc scrubbing			Sc stripping			precipitation and S/L separation	
stream no.		1	2	3	4	5	6	7	8	9	10	11	12	13	14	15	16	17	18	24	25
volume [L]		357	67	424	112	313	63	250	150	100	25	100	25	2.5	2.5	25	10	25	10	10	0.01
Sc	amount [g]	29 ± 1			8.5 ± 0.2	21 ± 1	6.1 ± 0.3	15 ± 1	1.9 ± 0.3	13.0 ± 0.4		0.10 ± 0.01	13 ± 1		0.005 ± 0.001	13 ± 1		0.10 ± 0.01	12.7 ± 0.4	0.500 ± 0.002	12.6 ± 0.16
	yield^b^ [−]				0.29	0.72	0.29	0.73	0.13	0.89		0.01	1.00		0.00	1.00	0.00	0.01	0.98	0.04	0.96
Ti	amount [g]	1900 ± 50			1600 ± 50	200 ± 10	200 ± 10	1.1 ± 0.1	0.4 ± 0.1	0.6 ± 0.1		0.4 ± 0.1	0.03 ± 0.01		0.006 ± 0.001	0.020 ± 0.002		n.d.	0.1 ± 0.1	0.110 ± 0.003	0.00 ± 0.01
	yield^b^ [−]				0.89	0.11	>0.99	0.01	0.32	0.58		0.67	0.05		0.20	0.67	0.00	0.00	5.00	1.00	0.00
Fe	amount [g]	11,300 ± 300			2100 ± 100	9000 ± 500	1700 ± 50	6300 ± 100	3500 ± 200	2900 ± 100		2200 ± 200	64 ± 9		n.d.	1.4 ± 0.3		n.d.	0.17 ± 0.05	0.110 ± 0.001	0.02 ± 0.01
	yield^b^ [−]				0.19	0.81	0.29	0.7	0.55	0.46		0.76	0.02		0.00	0.02	0.00	0.00	0.12	0.85	0.15
Zr	amount [g]	720 ± 30			740 ± 30	100 ± 5	89 ± 4	0.11 ± 0.01	0	0.11 ± 0.01		0.01 ± 0.01	0.12 ± 0.03		0.02 ± 0.01	0.11 ± 0.01		0.02 ± 0.01	0.09 ± 0.04	0.020 ± 0.001	0.080 ± 0.003
	yield^b^ [−]				0.88	0.12	>0.99	0.001	0	100		0.09	1.09		0.17	0.92	0.00	0.18	0.82	0.20	0.80
Th	amount [g]	41 ± 1			39 ± 4	8 ± 1	5 ± 1	2 ± 1	0.03 ± 0.01	1.9 ± 0.2		0.2 ± 0.03	1.7 ± 0.4		0.05 ± 0.03	1.8 ± 0.5		0.2 ± 0.1	0.4 ± 0.1	0.020 ± 0.001	0.210 ± 0.006
	yield^b^ [−]				0.83	0.17	0.78	0.22	0.01	1.07		0.11	0.89		0.03	1.06	0.00	0.11	0.22	0.09	0.91
U	amount [g]	9.3 ± 0.2			9 ± 1	1.2 ± 0.2	1.9 ± 0.2	0.97 ± 0.03	0.71 ± 0.02	0.24 ± 0.01		n.d.	0.20 ± 0.05		0.02 ± 0.01	0.19 ± 0.04		n.d.	0.18 ± 0.03	0.080 ± 0.005	0.150 ± 0.007
	yield^b^ [−]				0.88	0.12	0.22	0.78	0.73	0.24		0.00	0.83		0.10	0.95	0.00	0.00	0.95	0.36	0.64
V	amount [g]	725 ± 8			160 ± 10	520 ± 20	110 ± 2	368 ± 7	193 ± 9	178 ± 5		140 ± 10	10 ± 2		9 ± 2	1.1 ± 0.3		0.3 ± 0.1	0.6 ± 0.1	0.280 ± 0.003	0.32 ± 0.01
	yield^b^ [−]				0.22	0.72	0.21	0.71	0.53	0.48		0.79	0.06		0.90	0.11	0.00	0.27	0.55	0.47	0.53
Al	amount [g]	1710 ± 70			480 ± 60	1410 ± 70	280 ± 10	910 ± 40	192 ± 7	900 ± 40		710 ± 20	22 ± 3		21 ± 2	0.3 ± 0.1		0.2 ± 0.1	0.09 ± 0.02	0.40 ± 0.02	0.10 ± 0.05
	yield^b^ [−]				0.28	0.82	0.20	0.64	0.21	0.99		0.79	0.02		0.95	0.01	0.00	0.67	0.30	0.80	0.20

a Stream numbers refer to the steps defined previously (details in Figure 1), with the key streams being the acid waste (1), the NF concentrate (9), the SX raffinate (11), the strip liquor (18), and the crystallized (NH_4_)_3_ScF_6_ product (25).

b Yield per stage was calculated based on inputs from the direct upstream.

### Advanced Filtration

#### Precipitation and Removal of Interfering Metals

The received TiO_2_ acid waste contained Sc (∼81 mg L^–1^) and more than 30 other elements up to multiple grams per liter (Table 1). Some of these elements disturb SX but precipitate at pH 1.5, while the majority of Sc is preserved in the solution.^3,10^

After pH adjustment and MF, the majority of Sc (72%) remained in the filtrate (stream 5, Table 1). This result was higher than during the bench-scale tests, where only 56% of the Sc was preserved.^10^ Regarding the impurities, similar to the bench-scale tests,^10^ with the hydroxide sludge (∼250 L, stream 4), interfering elements were effectively removed (Ti: 88%, Zr: 88%, Nb 88%, U: 88%, Th: 83%). S/L separation worked slightly better on the pilot scale, yielding a 74% filtrate recovery in comparison to 69% during the bench-scale experiments.^10^

**Figure 2:**
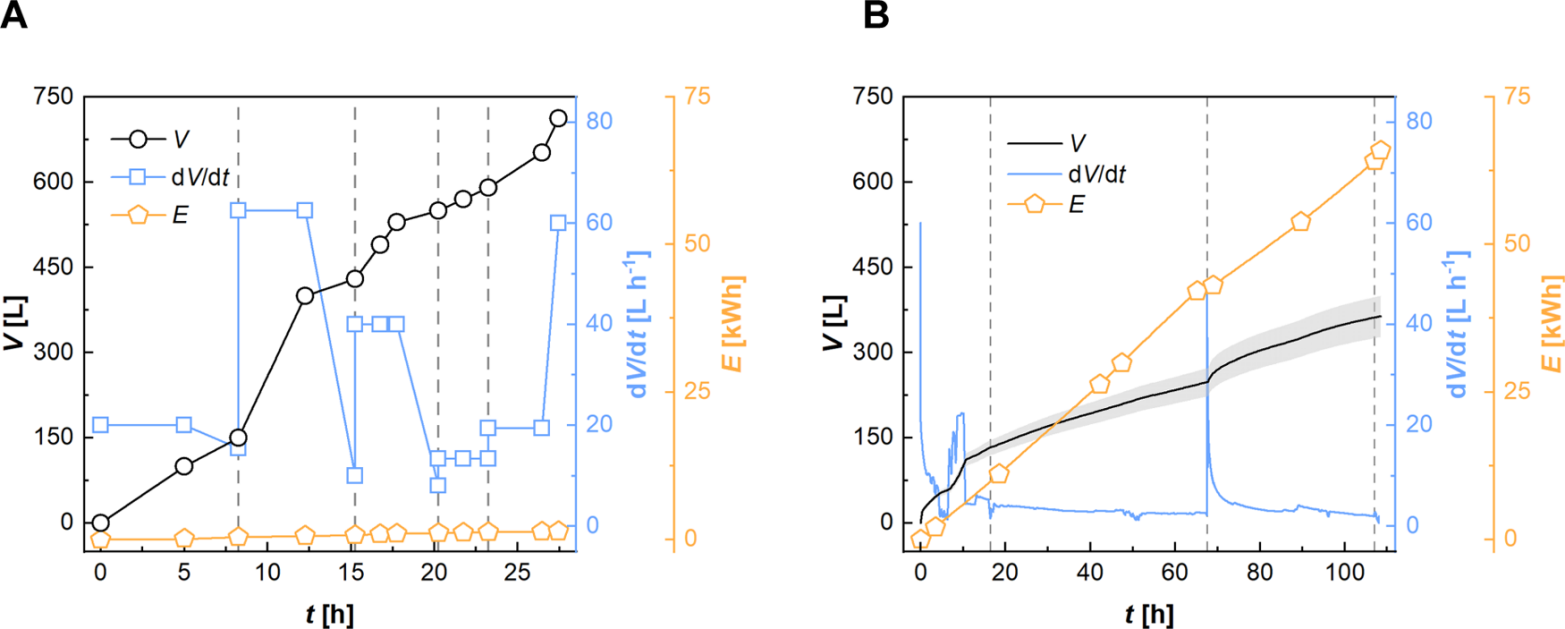
Volume of filtrate generated, flow rate, and energy consumption during microfiltration (A) and ultrafiltration (B). The gray dashed lines indicate the exchange of filter bags/ultrafiltration membranes.

The ratio between the sludge and bag filter volume changed disproportionately during upscaling. In the bench-scale tests, 1 L of sludge was removed using a bag filter of 1.9 L volume (ratio of 0.53).^10^ In the pilot phase, 250 L of sludge (stream 4) was separated using two bag filters of 17 L each (ratio of 7.4). As a result, the bag filters had to be emptied multiple times. The precipitate was allowed to settle for over 48 h, and the reactor was drained from top to bottom to prevent premature filter clogging. This strategy succeeded, as reflected in the initially higher filtration rates and longer operating intervals before the discharge of the filter cake than at the end of filtration (Figure 2A). However, the start-up phase (0–8 h) was exceptional, as the pump speed and immersion depth of the dip tube were not optimal, resulting in a low filtration rate and filter clogging after 150 L of filtrate was produced (Figure 2A).

Only a thickened sludge was obtained with no fully dewatered filter cake after MF. Therefore, the hygroscopic nature of the precipitated hydroxides impeded S/L separation. Flushing with pressurized air helped recover more filtrate but did not represent a satisfactory solution for continuous production. A plate filter press could help optimize the filtrate yield through higher compression and ease the procedure through automated discharge of the separated precipitate.^25^

The obtained filtrate was still partially turbid, being especially visible after filter exchange. This could be due to the use of extremely coarse filter bags, whereby the particle removal efficiency is usually low before the build-up of a filter cake. Felt bags with a nominal filter rating of 1 μm were used in this process. This means that particles of 1 μm and larger are retained but to an undefined percentage, as indicated by the manufacturer.^26,27^ For future tests, filtration materials with a 1 μm absolute rating (i.e., assured removal rate of >99% for particulates of ≥1 μm) could achieve a better separation result.

SEC for MF was ∼2.1 kWh m^–3^ of filtrate (Figure 2A), similar to the SECs reported for the MF of slurries, such as using a rotating MF (4 kWh m^–3^).^28^

Following MF, the filtrate was further clarified using UF. In this process, a 0.4 m^3^ ultrafiltrate (stream 7) was obtained, containing 73% of the Sc from the 0.5 m^3^ MF permeate (stream 5) (Table 1 and Figure 2B). Multiple elements were effectively removed with the residual suspended particles, including Ti (>99%), Zr (>99%), Nb (>99%), and Th (78%) (Table 1).

Directly after the deployment of new spiral wound elements, high filtration rates (40–60 L h^–1^/170–260 L m^–2^ h^–1^) were observed during UF. However, these rates decreased to <10% of their initial value within 5 h of operation. Rinsing with diluted hydrochloric acid did not restore permeability (tested after 16 and 107 h). After ∼109 h, an 80% permeate recovery was achieved, and UF was stopped because the feed had considerably thickened and the permeate flow had irreversibly decreased to below 2 L h^–1^.

Owing to the low filtration rate (average 3.6 L h^-1^/16 L m^–2^ h^–1^), the SEC for permeate production was high, eventually reaching 165 kWh m^–3^. Despite taking the challenging nature of the feed into account, the SEC appears to be at least an order of magnitude higher than the typical values reported for UF.^29,30^ Apparently, the spiral wound elements were rapidly clogged, which drastically affected their performance. Nevertheless, the UF was continued to provide feed for downstream NF experiments. This approach, however, was not cost-effective. High particle loading in the UF feed should be avoided through better S/L separation upstream of the UF to improve the operation. In this regard, employing a filter press (as used at the TiO_2_ manufacturing facility) or drum centrifugation would be recommended.^31^ In addition, different membrane designs could ease cleanability, allowing the recovery of lost permeability, thereby keeping the filtration rates high and increasing the membrane life span. In this process, capillary or tubular membrane elements should be tested.^25,32^ If the permeate flux is kept in the measured starting range of 170–260 L m^–2^ h^–1^, the SEC can be reduced by up to 95%. The suggested changes for MF and UF should result in higher Sc yields (currently 53%, three stages), thereby boosting the overall process efficiency.

#### NF

The pilot NF was based on bench-scale tests, aiming for a 60% permeate recovery (i.e., a final concentration factor of 2.5 (eq S1)).^10^ The targeted amount of NF concentrate (100 L) was set to allow downstream pilot SX. Thus, five batches of ultrafiltrate (50 L each) were concentrated with the same 2540 spiral wound membrane elements.

**Figure 3:**
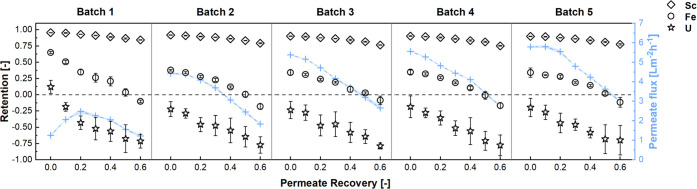
Element retentions and permeate fluxes during the five batches (each 50 L) of nanofiltration.

Sc retention during the pilot experiments was similar to that of the bench-scale tests.^10^ Starting at 0.96, a slight decrease to 0.85 after a 2.5-fold concentration was observed in the first batch (Figure 3). Sc retention was slightly lower in batch two, with initial and final values of 0.92 and 0.79, respectively (Figure 2A). The retention in batches three to five seemed to have reached constant values, being in each case initially 0.90 and 0.76 after a 60% permeate recovery (Figure 3). The measured Sc retention over the whole NF and all batches combined was 0.90, leading to a total Sc recovery yield of 87% (stream 9, Table 1), which was slightly higher than that reported for the bench-scale tests (84%).^10^ Overall, the Sc concentration was increased by a factor of ∼2.2 (from 60 to 130 mg L^–1^; Table 1).

Some impurities were successfully depleted by NF, such as Fe (−55%), V (−53%), or U (−73%) (Table 1). For instance, Fe retention was > 0.60 at the beginning of batch one and drastically decreased over the course of the NF, reaching negative values (Figure 3), that is, the permeate concentration was higher than the concentration in the retentate. From batch two onwards, the initial Fe retention was <0.40 and showed a falling trend during NF (Figure 3). The mean Fe retention over the entire NF stage was only ∼20%. Overall, the Sc over Fe selectivity (i.e., the ratio of *R*_Sc_/*R*_Fe_) was exceptionally high, reaching a mean of 4.5, whereas the bench-scale test reached a maximum of 2.7.^10^

Apart from Fe, U retention was remarkably low and constantly negative throughout the NF, except for the very first recorded value in batch one. The extremely high U permeability was reflected in an average retention of −0.97 and a yield of only 24% in the NF concentrate. This behavior was only matched by monovalent cations, such as Na^+^, reaching an average retention of −0.72 and a final yield of 29% in the NF concentrate. The U retention found is in line with the results of Remmen et al.^3^ One explanation could be the speciation of U in chloride-rich acidic environments, that is, the presence of monovalent or uncharged complexes. This finding was confirmed by EXAFS measurements showing the presence of chloro-uranyl complexes, such as UO_2_(H_2_O)*_x_*Cl^+^ and UO_2_(H_2_O)*_x_*Cl_2_, at HCl concentrations of ≥4 mol L^–1^.^33^ For future recovery of U from complex streams, where coextraction represents a challenge in SX, the aforementioned phenomenon in NF could be leveraged as a U preseparation step.

A steady increase in permeate flow during Sc concentration was observed from batch one to batch five (Figure 3). Except for the first batch, the permeate flow started at its highest value and declined as the feed concentration advanced. However, the permeate flux in the first batch started low (1.25 ± 0.05 L m^–2^ h^–1^), subsequently increased (2.5 ± 0.1 L m^–2^ h^–1^ at a 20% permeate recovery) and then decreased again (1.22 ± 0.07 L m^-2^ h^–1^ at a 60% permeate recovery) (Figure 3). This “parabolic” behavior was not previously observed in bench-scale tests. One explanation might be the five times larger membrane area in the pilot trials, which would have required a longer swelling time initially.^34^

During the bench-scale tests, membranes were only used once for the concentration experiments. As shown in this process, reusing was beneficial in terms of permeate flux and Sc selectivity over several impurities, such as Fe, V, or U (Figure 3). The behavior is in agreement with previous studies showing that acid soaking may result in higher permeability of polyethyleneimine-coated thin film composite membranes.^35,36^ Although not disclosed, AMS patents suggest a comparable active layer in the NanoPro A-3014 membrane.^37,38^ In addition, despite the higher permeate flux in batches three to five, no higher element retention was observed (Figure 3), indicating that the ion flux increased proportionally to the water flux (convective flow). Lopez et al. observed a similar behavior when testing NF for rare earth element recovery from acidic solutions and interpreted it as a sign of increased pore size caused by degradation.^39^ In contrast to the aforementioned study, an especially acid-resistant NF membrane was used in our study to withstand HCl exposure. Although partial membrane degradation cannot be excluded, the similarity of element retentions and permeate fluxes in batches three to five indicates the NanoPro A-3014′s primary suitability for the application (Figure 3). Therefore, the membrane can be further reused. Based on the results, longer membrane equilibration prior to NF should be considered for future Sc recovery.

The production of 2.5-fold concentrated acid waste through NF took 31 h (310 h m^–3^). The increase in the permeate flow rate (average batch one: 1.8 L m^–2^ h^–1^; average batch five: 4.7 L m^–2^ h^–1^) resulted in a decreased operating time with each batch. Furthermore, the energy consumption rate was almost constant during the entire NF (1 kWh h^–1^). Accordingly, the respective energy cost decreased with each batch due to the accelerating filtration rate. The mean SEC for concentrate production was 327 and 265 kWh m^–3^, considering only the last three batches (both referring to concentrate volume). The key to the high energy demand of NF was the low permeate flux (max. 5.8 L m^–2^ h^–1^ at 35 bar TMP). The use of RO or NF with small membrane permeability has already been reported (e.g., in the field of acid purification).^40^ However, SEC needs to be optimized to improve process profitability for future applications. In this process, highly permeable LbL membranes could be of interest as soon as more stable products suitable for highly concentrated streams become commercially available.^3^ The minimization of the cross-flow rate could be an option in the case of the NanoPro A-3014. A reduction is possible as long as permeate flux and Sc retention are not impaired^41^ and no scaling occurs (unlikely at pH 1.5). Moreover, the energy demand per membrane area can be decreased by further upscaling the system.^42^ For example, a pump delivering 10 times the flow would consume proportionally more energy but could feed an 8040 element that has 15 times the membrane area of a 2540 element.^43−45^ Consequently, SEC could be cut by a third. Furthermore, a smaller spacer (31 mil instead of 46 mil) could increase the membrane area per element, specifically by 25%, in the case of 8040 elements.^43^ Finally, the implementation of energy recovery devices, such as Pelton turbines, could recover 30–40% of the total energy.^46,47^ These adjustments could result in 70% savings in SEC.

In summary, the pilot NF performed better than the bench scale, recovering more Sc with better selectivity and demonstrating that membrane reuse does not only reduce investment costs but also improves Sc selectivity and operating speed. The high SEC calls for membrane and system optimization, providing a starting point for future efforts.

### Solvent Extraction

**Figure 4:**
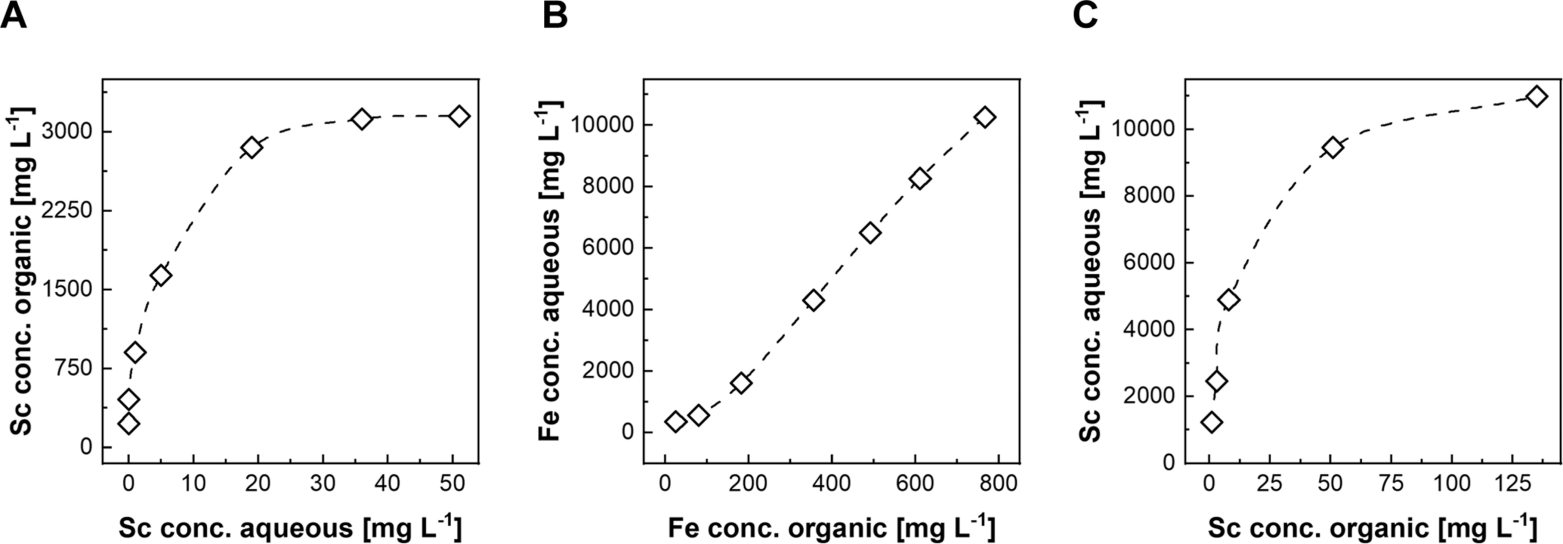
Equilibrium diagrams for loading (A), scrubbing (B), and stripping (C) of Sc using 0.2 mol L^–1^ of D2EHPA with 0.05 mol L^–1^ of N1923 in D80 kerosene at the bench scale.

As previously reported, a synergistic mixture of D2EHPA and N1923 reduces the coextraction of impurities, such as V and Fe, in the SX circuit.^10^ Based on this, 0.2 mol L^–1^ of D2EHPA with 0.05 mol L^–1^ of N1923 in D80 kerosene was used as the organic solution for the pilot testing. The equilibria for Sc loading, scrubbing, and stripping were determined on a bench scale prior to the pilot experiments (Figure 4). A maximum loading of 3 g L^–1^ was observed during the laboratory investigations (Figure 4A). However, worse separation behavior was observed beyond the Sc loading of 1.5 g L^–1^. The organic started foaming, slowing down phase separation due to high Sc loading, which ultimately prevented continuous processing at this loading level. Therefore, Sc loading was chosen between 0.5 and 0.7 g L^–1^, eliminating phase separation issues and yielding fast separation. Based on the equilibrium loading diagram, full Sc loading required two stages of extraction (Figure 4A).

In terms of scrubbing behavior, using HCl (4 mol L^–1^) in 3–4 scrubbing stages resulted in the effective removal of the coextracted Fe from the loaded organic (Figure 4B). In addition, D2EHPA showed considerably higher affinity to Fe^3+^ than to Fe^2+^, wherefore the addition of Fe^0^ suppressed Fe coextraction by reducing Fe^3+^.^10^ As such, Fe^0^ was added to the NF concentrate before the loading stage. The scrub liquor (i.e., spent HCl after scrubbing) was recycled into the loading feed solution to eliminate Sc losses and lower the solution pH, thereby suppressing Fe^2+^ coextraction.

Investigations on Sc stripping equilibrium (Figure 4C) confirmed the effectiveness of NH_4_F (3 mol L^–1^). Complete Sc stripping was achieved in most cases. However, the solubility limit of (NH_4_)_3_ScF_6_ (∼7.5 g L^–1^)^19^ at Sc concentrations above 2 g L^–1^ was exceeded, leading to crystallization. Since solid precipitate could harm the SX process by forming cruds and inseparable phases, causing organic losses, a final Sc concentration of 1.0–1.5 g L^–1^ was targeted for the strip liquor. Based on the equilibrium data, four stages of stripping were required for effective Sc stripping (Figure 4C).

The settling behavior in each SX step was investigated, and the separation speeds of the aqueous and organic solutions were calculated (Table 2). In all cases, separation speeds exceeded 2 m h^–1^, implying rapid, successful separations (Table 2). Moreover, both mixing modes (aqueous or organic phase as the dispersant) were tested. However, no impact on separation behavior was observed. Generally, no phase separation problems occurred in the pilot SX tests.

**Table 2:** Average Phase Separation Speed in Each Step of SX

process step	separation speed [m h^–1^]
extraction	3.4
scrubbing	2.6
washing	14.8
stripping	10.8
conditioning	3.9

The processing of the entire NF concentrate (100 L) lasted for 17 h. The pilot SX worked efficiently with only minute Sc losses, reaching a yield of ∼98% (three stages) and a 10-fold increase of Sc concentration in the strip liquor (∼1.27 ± 0.04 g L^–1^; stream 18; Table 1). Impurities in the product included V, Th, U, and Fe (Table 1). Despite the removal of most Fe, minute amounts were still present in the strip liquor, probably due to the spontaneous oxidation of Fe^2+^ to Fe^3+^ during the continuous operation. To prevent this occurrence in the future, sealed mixer-separator units could be used instead of running the SX in an open atmosphere. Although only traces of Th and U were observed in the NF concentrate, they were almost inseparable from Sc in SX. Therefore, 75% of U and 21% of Th ended up in the strip liquor. Notably, the mass balance for Th after stripping did not add up, and 67% of the total extracted Th was neither measured in the stripped organic nor in the strip liquor. Insoluble Th complexes possibly formed after NH_4_F addition and precipitated without being noticed in the pilot unit. In the case of V, coextraction was well suppressed by the use of N1923 as coextractant, leading to only 6% coextraction (Table 1). In the scrubbing stage, 90% of the extracted V was removed (Table 1). The 0.3% (i.e., 0.6 g) initial V that was eventually stripped still made it a major impurity in the strip liquor due to its high starting concentration (Table 1). In total, approximately 90% Sc purity was reached, which was below par with the previously reported bench-scale result of 97%.^10^ Although the entire NF concentrate was processed, the SX process had probably not yet reached its equilibrium. Supposedly, higher purities can be attained in a longer continuous operation. During the pilot trials, the purity levels in the samples collected increased as the SX process continued.

### Antisolvent Crystallization

**Figure 5:**
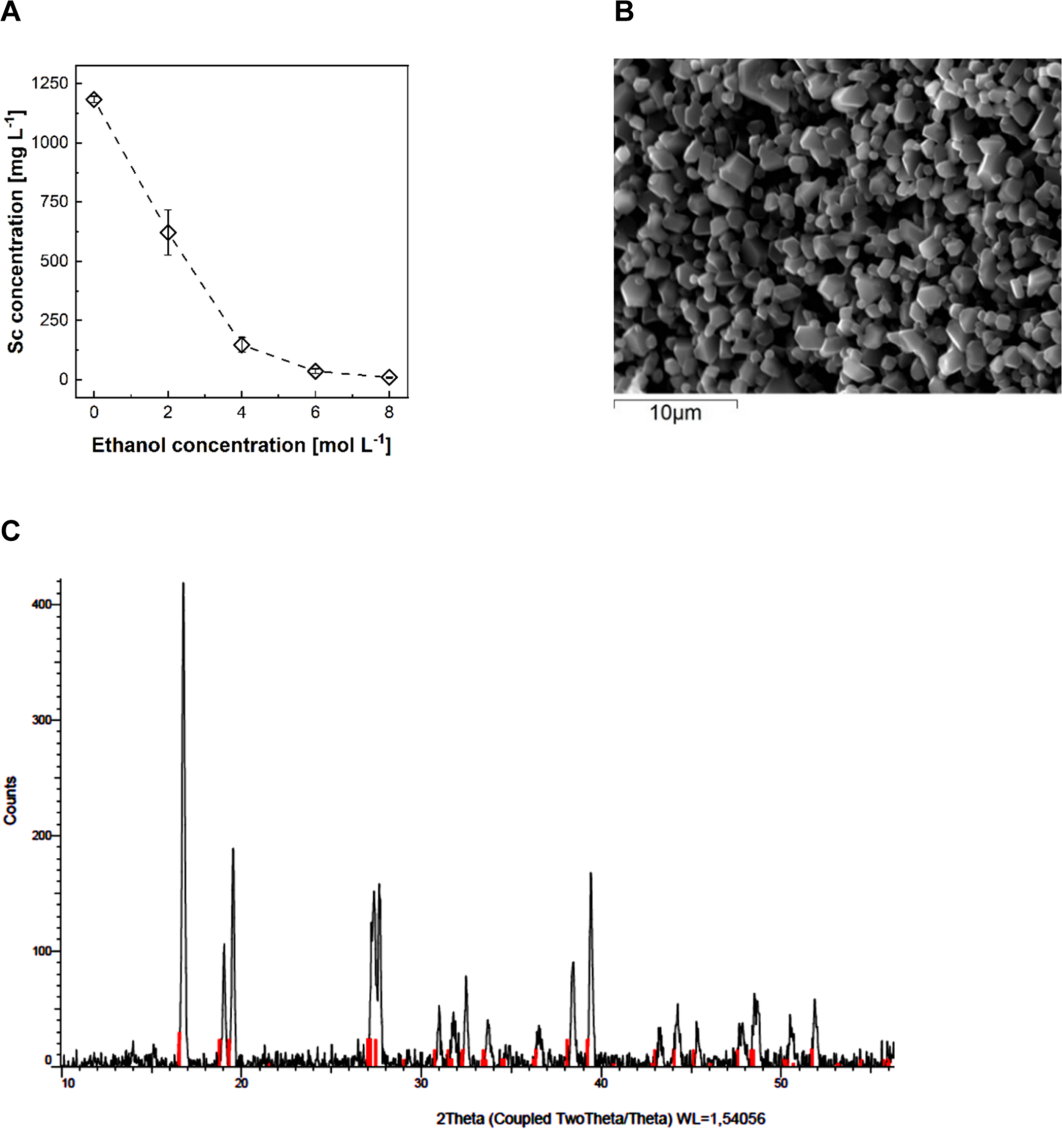
Solution concentration profile for Sc (A), SEM micrograph of the solid obtained at 8 mol L^–1^ of ethanol (B), and XRD pattern of the solid product obtained at 8 mol L^–1^ (C). The red lines are the reference pattern for (NH_4_)_3_ScF_6_ of monoclinic-structure PDF card 00-040-0595 (C).

Sc crystallization started quickly after the addition of ethanol to the strip liquor. The amount of precipitated Sc asymptotically approached a scandium recovery of > 95% with increasing ethanol concentration (Figure 5A). A concentration of 8 mol L^–1^ appeared optimal to maximize Sc recovery with a yield of 96% (Table 1).

After instantaneous antisolvent addition (8 mol L^–1^), discrete, regular-shaped crystals with an average size of approximately 1–2 μm were obtained, as seen in the SEM image (Figure 5B). The mean size and size distribution of the crystal product can be controlled by seeding and supersaturation control.^20^ Powder XRD measurements identified the obtained solids as predominantly (NH_4_)_3_ScF_6_ (PDF 00-040-0595; Figure 5C). The peaks of other ammonium metal fluorides, such as Zr, V, Al, or Fe, which were present in the strip liquor, could not be detected. This could indicate low concentrations in the solid material but may also be attributed to similar peak positions of most ammonium metal fluorides.

Element concentrations were also measured in the strip liquor before and after ASC with 8 mol L^–1^ of ethanol (Table 1). Based on the results, the solid product’s purity was determined to be 93.5% on a metal basis or 95.1% on the basis of ammonium metal fluorides (Table S1; assuming the formation of ammonium metal fluoride complexes for all impurities). Impurities could be incorporated into crystal lattices or adhere to the crystal surfaces without actually precipitating as ammonium metal fluorides. As reported previously, Ti tends to remain solubilized, most likely due to its stable titanyl ion (TiO^2+^) in the solution.^18^ Similar to their abundance in the strip liquor, the major impurities found in the solid product were V, Th, and U (ordered by mass fraction; Table 1). Furthermore, minute amounts of Al and Zr are present in the solid (Table 1). Comparable to SX, the product purity after ASC was below par compared with the previously reported purities of ca. 99%.^17^ As previously described, SX was probably further away from its equilibrium than during the bench-scale tests, which also negatively affected the downstream ASC. Hence, the easiest solution would be to further optimize Sc selectivity upstream to ASC. This result could also be partly due to the lower initial Sc concentration in the strip liquor than previously reported.^17^ Nonetheless, crystallization in more stages, starting with a lower amount of antisolvent and better control of the supersaturation during crystallization, could help increase the purity, potentially at the cost of total yield.^18,20,48^ Moreover, purification of the product could be achieved through a combination of SX and ion exchange.^23,49^

In terms of ASC process design, the required ethanol amount of 0.88 L per liter strip liquor appeared high. However, the spent ethanol can be distilled and reused in ASC without deterioration of precipitation efficiency. In a previous study, methanol and ethanol recovered through simple distillation with alcohol purities of 75–85% (v/v) showed Sc recovery efficiencies > 97% when reused in ASC.^50^ Furthermore, after antisolvent distillation, the spent aqueous solution, which was partially depleted in NH_4_F, can be reused in the SX stripping stage with adequate make-up (Figure 1).

### Process Flows and Production Cost Assessment

The developed process was benchmarked based on the production of 1 kg of ScF_3_ as the marketable product closest to (NH_4_)_3_ScF_6_. As previously reported, (NH_4_)_3_ScF_6_ can be easily converted into ScF_3_ by calcination.^51^ Following previous studies, the conversion of 2.1 kg of (NH_4_)_3_ScF_6_ into 1 kg of ScF_3_ was considered with an input of 0.9 kWh electricity (Table 3).^52−54^ Furthermore, for AF, filter materials, such as the organic phase in SX, were assumed to be fully reusable. For SX and ASC, 90% recyclability of NH_4_F solution and antisolvent was assumed, respectively. The underlying prices used for assessment are given in the SI (Table S2).

**Table 3:** Energy and Material Flows and Costs to Produce 1 kg of ScF_3_

description		AF	SX	ASC	CAL	total	sum energy and material costs [€]
acid waste	[kg]	13′198				13′198	
ethanol	[kg]			25		25	6–20
HCl 33%	[kg]		42			42	4-5
NaOH 30%	[kg]	3′160				3′160	230–253
NH_4_F (3 mol L^–1^)	[kg]		36			36	4
Fe powder	[kg]		5.3			5.3	10–12
water	[kg]		69			69	0.01
electricity	[kWh]	2′426	3.7	1.1	0.9	2′432	119–136
heat	[kWh]			576		576	12
waste	[kg]	13′198	4′012	42		17′252	
total costs	[€]	350–389	18–22	18–31	0.04–0.05	386–442	386–442

The total input for the production of 1 kg of ScF_3_ from ∼13,000 kg of AW totaled ∼3400 kg of materials and ∼3000 kWh of energy consumption (Table 3). The generated waste was ∼17,300 kg. Given that the waste is a mix of hydroxides, it could be disposed of similarly to the TiO_2_ plant waste treatment.

**Figure 6:**
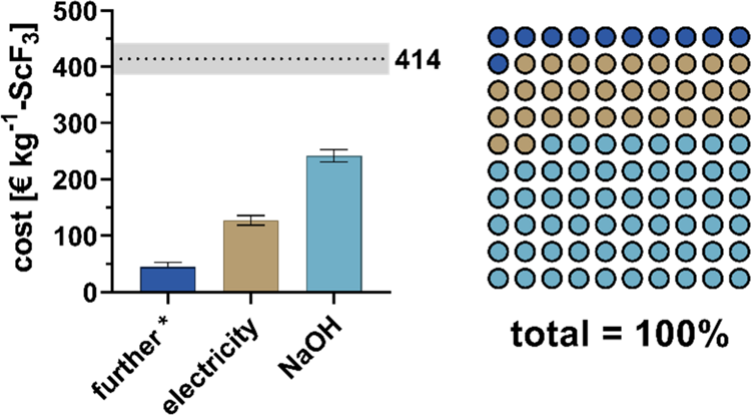
Costs for ScF_3_ production. Bars indicate maximal/minimal assumptions for prices. Note that ethanol, HCl, NH_4_F, Fe powder, water, and heat combined contributed little (11%) to the overall cost and are summarized as “further”.

The total material and energy costs to produce 1 kg of ScF_3_ were ∼414 ± 28 € (Table 3 and Figure 6). United States Geological Survey (2022) reported a price (1–5 g lot size) of US$216,000 (∼216,000 €) per kilogram of ScF_3_, which is assumed to be very high.^5^ Prices on online portals (e.g., alibaba.com) range between ∼721 and 1546 € kg^1^ of ScF_3_ (99–99.99% purity; Table S2). Hence, the production costs for ScF_3_ determined in this study were considerably lower than the market prices reported.

Among all of the process inputs, NaOH had the highest cost share (∼58%; Figure 6), followed by electricity consumption (∼31%; Figure 6). All further inputs contributed only 11% to the total costs (Figure 6). On the process level, the initial AF step had the major cost share (∼89%; Table 3). Therefore, process optimization should target the AF stage first. As previously described, a high optimization potential for the energy consumption of AF is expected (savings of 95% for UF and 70% for NF). This could reduce the energy consumption by ∼2000 kWh kg^–1^ of ScF_3_, lowering the production cost by 25% or 110 € kg^–1^. The primary cost driver would still be neutralizing with NaOH. The neutralization, although assigned to the AF stage in this study, is already a part of waste treatment in TiO_2_ production. Hence, the actual cost of pH adjustment in AF should be calculated as the difference between the cost for neutralization with NaOH or with lime/limestone, similar to the current practice. Using CaO/CaCO_3_ would not be an option in AF, as Ca^2+^ shows considerably higher retention than Na^+^ in NF, thereby increasing the osmotic pressure and deteriorating filtration performance. Kapil et al. compared the neutralization efficiency for different chemicals, revealing a 10% lower consumption of CaCO_3_ compared with NaOH for reaching the same pH.^55^ Thus, considering a slightly lower price per kilogram for limestone than for caustic soda, a treatment with NaOH is expected to cost roughly 20% more (Table S3). This means that the existing TiO_2_ production already covers 80% of the neutralization costs (i.e., ∼200 € kg^–1^ ScF_3_) previously allocated to AF. Therefore, the additional neutralization cost during AF is estimated to be 50 ± 5 € kg^–1^ ScF_3_.

In summary, the entire AF would realistically cost around 70 ± 30 € kg^–1^ ScF_3_, which is approximately 80% lower than the current pilot operation. In this scenario, a total material and energy cost for ScF_3_ of 120 ± 40 € kg^–1^ is conceivable. The overall process yield (43%, nine stages) could be improved, bearing the potential to cut the production cost in half. The Sc losses during the initial S/L separation (MF and UF) could be easily minimized by exchanging bag filtration with a filter press similar to that used in TiO_2_ production.

## Conclusions

This study demonstrated the feasibility of combining AF techniques, SX, and ASC to obtain 95% pure (NH_4_)_3_ScF_6_ as a close-to-market Sc product from a real TiO_2_ acid waste. Major challenges during AF included the low filtration rates because of the small particle size and hygroscopicity of the precipitated hydroxides and the osmotic pressure of the feed. NF improved with the progression of the pilot tests, yielding higher permeate flux and Sc selectivity, which were interpreted as benefits of membrane equilibration. Overall, the process volume was reduced through NF by 60%, with 87% Sc yield and depletion of impurities such as Fe, V, and U.

During pilot SX, high separation efficiency for Sc was observed with the previously published process.^10^ Phase separation worked rapidly, and tertiary phases did not occur. However, the achieved purity still left room for improvement, calling for longer test runs that allow for better process equilibration and a closed system to minimize spontaneous Fe oxidation. In total, a 10-fold concentrated Sc liquor (98% yield, three stages) was produced, with minimal coextraction of competing elements such as Fe or V.

Using strip liquor, the ASC tests indicated the addition of 0.88 v/v ethanol as the best option, delivering the highest Sc yield (96%).

The overall process has the potential to produce ScF_3_ at competitive market prices from a European secondary source. Thus, the combination of AF-SX-ASC could boost the supply of Sc, mitigating possible policy-induced shortages in the future.

## Supporting Information Available

Equations used to calculate the advanced filtration performance, overview of the pilot plants (AF and SX), breakdown of elemental concentrations used for purity calculations, and overview of specific costs used for the production cost assessment (PDF)
