# Supplementary material for: From Trace to Pure: Pilot-Scale Scandium Recovery from TiO2 Acid Waste
Source: ACS Sustain Chem Eng. 2023 Apr 6;11(15):5883–94. doi: 10.1021/acssuschemeng.2c06979 (PMC10114082; doi:10.1021/acssuschemeng.2c06979)
Supplement: Supplementary file 1 — sc2c06979_si_001.pdf [file sc2c06979_si_001.pdf]

Supporting information

From trace to pure: pilot scale scandium recovery from TiO_2 acid waste

Sebastian Hedwig, Bengi Yagmurlu, Edward Michael Peters, Victor Misev, Dirk Hengevoss, Carsten Dittrich, Kerstin Forsberg, Edwin C. Constable, Markus Lenz*

*corresponding author

Number of pages: 6

Number of figures: 2

Number of tables: 2

Advanced filtration

Calculations

The dimensionless concentration factor (X) was calculated as follows:

$$X = \frac{V_{\text{feed}}}{V_{\text{concentrate}}} \tag{S1}$$

with the feed volume (V_{feed}) and concentrate volume ($V_{\text{concentrate}}$).

The dimensionless retention (R_{M}) of an element (M) in NF was calculated as follows:

$$R_{\text{M}} = 1 - \frac{c_{\text{M,permeate}}}{c_{\text{M,feed}}} \tag{S2}$$

with the concentration of an element in the permeate ($c_{\text{M,permeate}}$) and in the feed ($c_{\text{M,feed}}$).

The permeate flux (J_{permeate}) in L m^{-2} h^{-1} was calculated as follows:

$$J_{\text{permeate}} = \frac{V_{\text{permeate}}}{A_{\text{membrane}} \cdot t} \tag{S3}$$

with the permeate volume (V_{permeate}) in litre, the time in hours (t) and membrane area in m^2 (A_{membrane}).

The specific energy consumption (SEC) in kWh m^{-3} was calculated as follows:

$$\text{SEC} = \frac{W_{\text{electric}}}{V_{\text{product}}} = \frac{P_{\text{electric}} \cdot t}{V_{\text{product}}} \tag{S4}$$

with the electric work W_{electric} in kWh and the product volume (V_{product}) in m^3.

Overview pilot plant

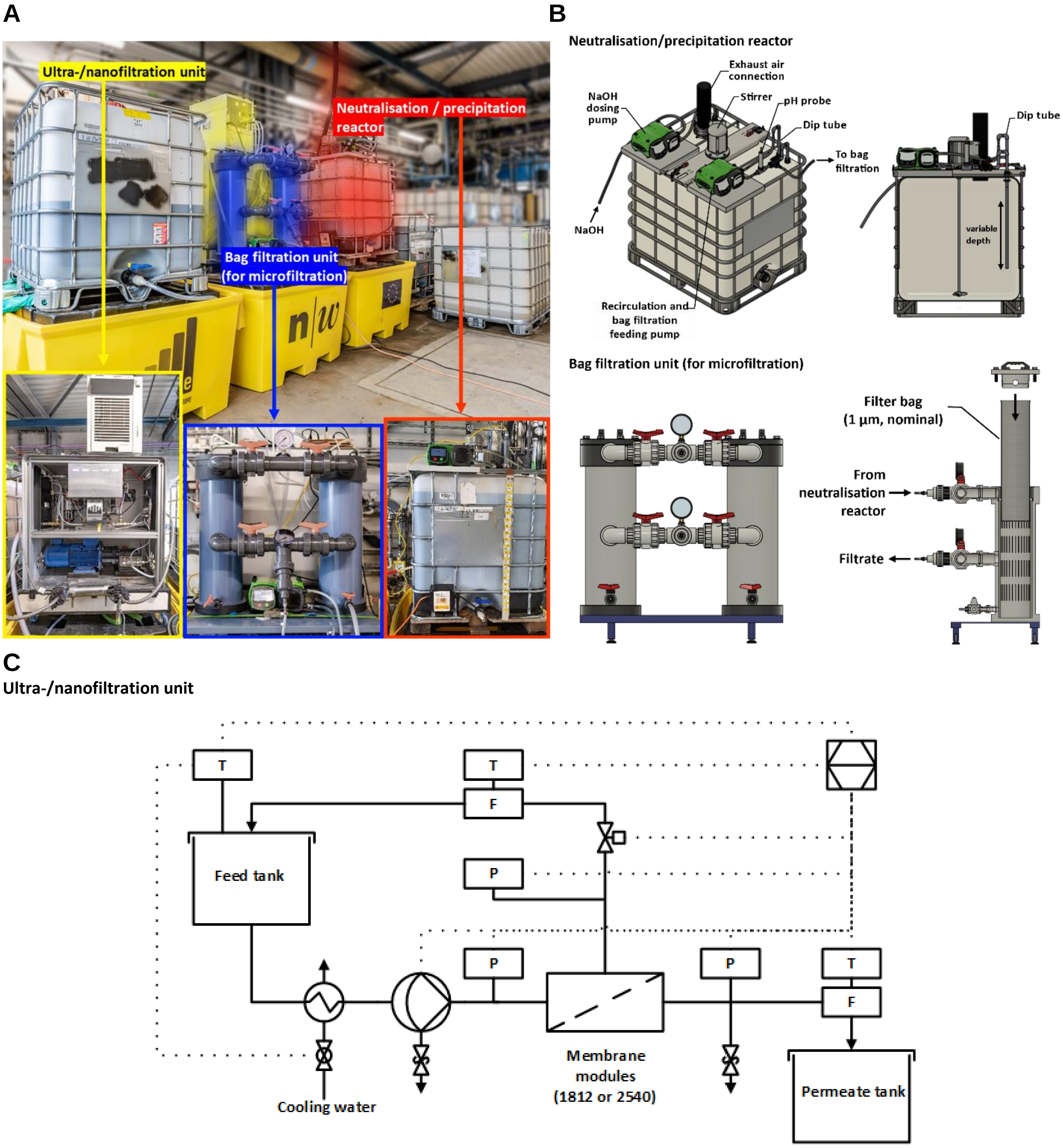

Figure S1: Overview of the advanced filtration pilot unit (A), including computer aided design drawings of the neutralisation reactor and the bag filtration unit (B) and a piping and instrumentation diagram of the ultra- and nanofiltration unit (C).

Solvent extraction

Overview pilot plant

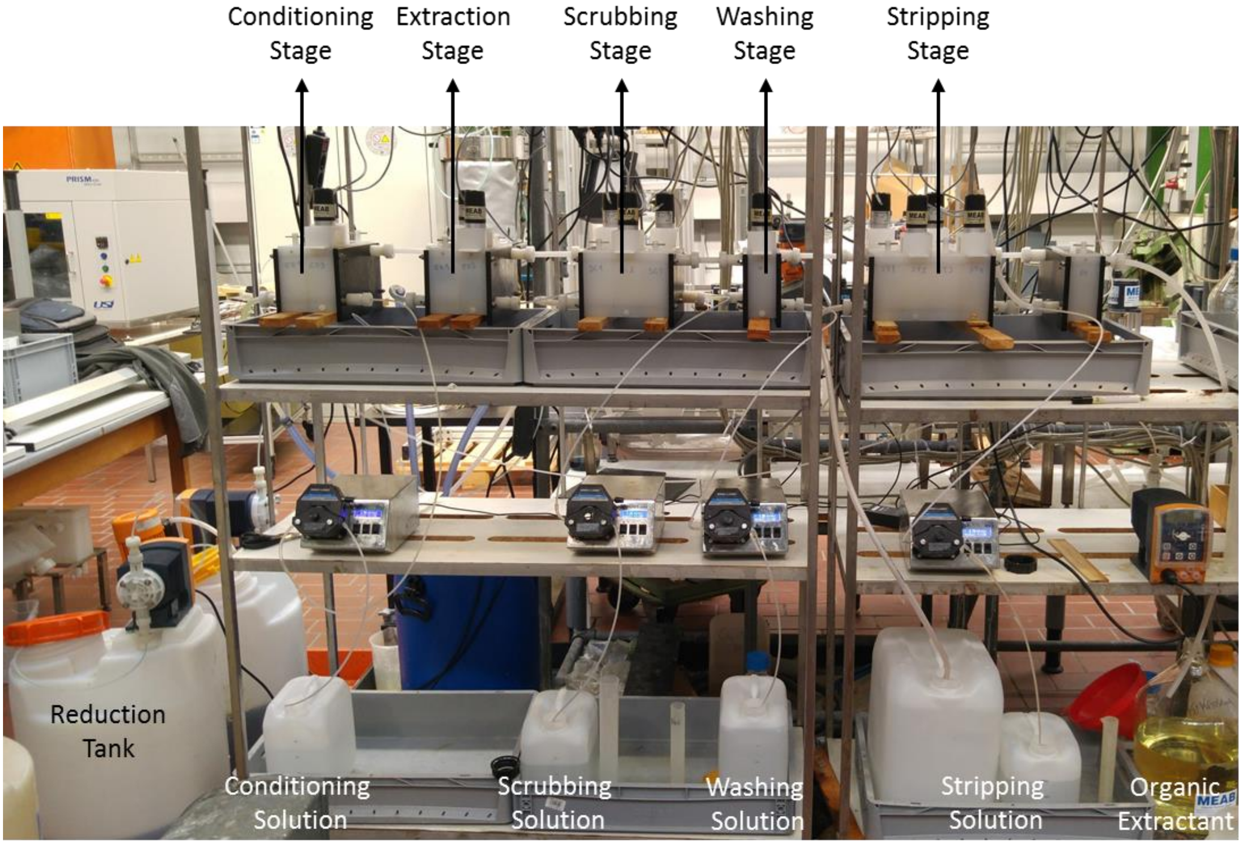

Figure S2: Overview of the solvent extraction pilot unit.

Anti-solvent crystallization

Table S1: Amounts of metals in the strip liquor (10 L) before and after precipitation. Precipitated amounts were calculated as the difference between ‘before ASC’ and after (‘in solution’). Purities were calculated as the mass fraction of Sc, resp. $(NH_4)_3ScF_6$ in the total amount of metal, resp. $(NH_4)_xM^{y+}F_{(x+y)}$. Values are averages of triplicates with their associated standard deviations.

		Before ASC	In solution	Precipitated	As $(NH_4)_xM^{y+}F_{(x+y)}$
**Sc**	**Amount [g]**	13.09	0.5 ± 0.002	12.6 ± 0.16	59.64
	**Yield* [-]**		0.04 ± 0.01	0.96 ± 0.01	
**Ti**	**Amount [g]**	0.11	0.11 ± 0.003	0 ± 0.01	0.00
	**Yield* [-]**		1 ± 0.04	0 ± 0.04	
**Fe**	**Amount [g]**	0.13	0.11 ± 0.001	0.02 ± 0.01	0.08
	**Yield* [-]**		0.85 ± 0.02	0.15 ± 0.02	
**Zr**	**Amount [g]**	0.10	0.02 ± 0.0005	0.08 ± 0.003	0.24
	**Yield* [-]**		0.2 ± 0.01	0.8 ± 0.01	
**Th**	**Amount [g]**	0.23	0.02 ± 0.001	0.21 ± 0.006	0.35
	**Yield* [-]**		0.09 ± 0.01	0.91 ± 0.01	
**U**	**Amount [g]**	0.23	0.08 ± 0.005	0.15 ± 0.007	0.29
	**Yield* [-]**		0.36 ± 0.03	0.64 ± 0.03	
**V**	**Amount [g]**	0.60	0.28 ± 0.003	0.32 ± 0.01	1.37
	**Yield* [-]**		0.47 ± 0.01	0.53 ± 0.01	
**Al**	**Amount [g]**	0.47	0.4 ± 0.02	0.1 ± 0.05	0.72
	**Yield* [-]**		0.8 ± 0.04	0.2 ± 0.04	
**Purity (%)**		87.5		93.47	95.12

Process flows and production cost assessment

Table S2: Energy and material flows to produce 1 kg ScF_3 by AF, SX, ASC and CAL. Specific energy and material costs.

Stream	Specific costs	Source
ScF_3 (99-99.99%)	721 – 1546 € kg^{-1}	https://www.alibaba.com/product-detail/Rare-earth-99-99-99-Scandium_1600274929720.html?spm=a2700.gallery-offerlist.normal_offer.d_title.7d1f4b99lzMQrZ&s=p
Ethanol	0.25 – 0.80 € kg^{-1}	alibaba.com
HCl 33%	0.09- 0.12 € kg^{-1}	Industrial partner
NaOH 30%	0.073 –0.08 € kg^{-1}	Industrial partner
NH_4F	1.04 € kg^{-1}	lanxess.com, alibaba.com https://www.alibaba.com/product-detail/Professional-export-factory-direct-sales-Ammonium_1600572543550.html
Fe powder	1.91-2.27 € kg^{-1}	https://www.alibaba.com/product-detail/China-Factory-Bulk-High-Pure-Atomized_62526820923.html?spm=a2700.gallery-offerlist.normal_offer.d_title.75d2561aspiX4N&s=p
Water	0.180 € m^{-3}	Industrial partner
NH_4F 3 mol L^{-1}	0.12 € kg^{-1}	
Electricity	0.049– 0.056 € kWh^{-1}	ec.europa.eu/eurostat/databrowser/view/NRG_PC_205__custom_1114276/default/table?lang=en
Heat	0.02 € kWh^{-1}	Industrial partner
$CaCO_3$	0.062 – 0.073 € kg^{-1}	https://www.alibaba.com/product-detail/White-Powder-Caco3-Powder-Calcium-Carbonate_10000003295317.html?spm=a2700.gallery-offerlist.normal_offer.d_image.3fb42725fIcYLj
